# Health Literacy Curricula in Pediatric Residency Programs: A National Survey

**DOI:** 10.3928/24748307-20240813-01

**Published:** 2025-01

**Authors:** Nicole Meyers, Suzanne Friedman, Andrea Morrison, Marguerite Costich, Aditi Gupta, Brittany Moore, Mary Ann Abrams, Margaret Wood, Joy Solano

## Abstract

**Objective:**

Using health literacy informed communication strategies can mitigate health inequities. Despite the high prevalence of limited health literacy among parents and children, pediatricians infrequently use clear communication techniques and further education is imperative. There is minimal literature exploring health literacy curricula in pediatric residencies. We sought to evaluate health literacy education across pediatric residency programs.

**Methods:**

The Academic Pediatric Association's Health Literacy Special Interest Group performed a national, cross-sectional survey of pediatric associate program directors from July 2022 to September 2022. We asked about the presence of health literacy curricula, teaching strategies used, content highlighted, outcomes assessed, and barriers encountered.

**Results:**

Among 55 associate program directors from programs diverse in setting, size, and geographic region who participated, most (84%) reported their residents would benefit from more health literacy training. Only 44% reported the presence of health literacy education, with minimal teaching of evidence-based principles, such as the universal precautions approach to clear communication.

**Conclusion:**

Health literacy is infrequently taught in pediatric residency programs and there is appreciable variability among existing curricula. There is need for greater dissemination of existing resources, as well as standardization of curricula and assessment tools to ensure pediatricians are well-versed in use of health literacy-informed communication techniques. [***HLRP: Health Literacy Research and Practice*. 2025;9(1):e3–e7.**]

Improving providers' ability to communicate with populations with limited health literacy is a Healthy People 2030 priority (Healthy People 2030, n.d.; Healthy People 2020, n.d.). Pediatric providers infrequently use health literacy informed communication techniques (Glick et al., 2023) despite the high prevalence of limited health literacy among populations they serve. Only 1 in 7 parents has proficient health literacy skills (Yin et al., 2009), and health literacy is a modifiable predictor of language (Yin et al., 2009) and racial and ethnic health disparities (Coleman et al., 2023).

## Background

Educating physicians on how to mitigate negative effects of limited health literacy is imperative. The Agency for Health Care Research and Quality (AHRQ) endorses several health literacy-informed communication strategies such as plain language, Teach Back, and chunking information (AHRQ, 2024) which have been promoted by experts (Coleman et al., 2017) and demonstrated beneficial effects on health outcomes (Griffey et al., 2015). Residency training is an opportune time to disseminate health literacy education (Glick et al., 2023; Sullivan, 2017); yet existing literature focuses on family medicine and internal medicine programs (Allenbaugh et al., 2019; Green et al., 2014; Sullivan, 2017). To our knowledge, only one study discusses teaching health literacy skills to pediatric residents, who have particular needs given the unique triad of provider, parent, and patient (Costich et al., 2022).

As part of a larger initiative to create a pediatrics-specific health literacy curriculum, the Academic Pediatric Association's Health Literacy Special Interest Group (APA Health Literacy SIG) conducted a national needs assessment to evaluate health literacy education within pediatric residency programs. We sought to describe the prevalence and characteristics of current health literacy education and barriers to implementation.

## Methods

### Survey Design and Distribution

We conducted a national, cross-sectional survey of pediatric associate program directors (APDs) to evaluate the state of health literacy education within pediatric residency programs. APDs were chosen as the survey audience due to their high-level perspective on our study objectives and greater availability to be surveyed compared with program directors. We adapted a survey from prior literature used to assess health literacy education in family medicine programs (Coleman et al., 2016). The survey, which had validity evidence in their populations (Coleman et al., 2016), underwent minor rewording for readability and applicability to pediatrics by referencing the caregiver in addition to the patient. Use of a previously published survey and review by the APA Health Literacy SIG ensured content validity evidence. Our survey included open-ended and multiple-choice questions on whether health literacy education is provided, educational strategies used, content highlighted, outcomes assessed, and barriers encountered. Additionally, we assessed how programs value health literacy education using Likert scale items. Our study was deemed exempt by the Institutional Review Board at Children's Mercy Kansas City.

We partnered with the Association of Pediatric Program Directors (APPD) Research and Scholarship Learning Community (RSLC) who distributed our survey to APDs using LimeSurvey (http://www.limesurvey.org) from July 2022 to September 2022. Per their protocol, a single APD from each program is surveyed, receiving one invitation and up to two weekly reminders. If there is no response, they move down the list alphabetically until one APD completes the survey from each program; if no APD completes it, the program is deemed a nonresponder. When the survey closed, the APPD provided de-identified, aggregated survey data and program demographics to study authors.

### Data Analysis

We analyzed quantitative results using descriptive statistics and performed chi-square analyses on respondent and non-respondent demographics. We used thematic analysis (Kiger & Varpio, 2020) to analyze the survey comments on barriers to implementing and maintaining health literacy education in residency. Two authors (B. M. & J. S.) familiarized themselves with the comments, manually generated codes and searched for early themes (Kiger & Varpio, 2020). The codes and developing themes were reviewed by a third author (N. M.) to facilitate discussion as we placed coded data within themes (Kiger & Varpio, 2020). Four iteratively refined overarching themes were then defined and named.

## Results

A total of 55 APDs from 193 programs completed our survey (28% response rate). This is higher than those of recent APPD RSLC surveys (2021 to 2022) of APDs (response rates 15% to 23%) (Association of Pediatric Program Directors, n.d.). We compared responding and non-responding institutions' demographics and found no differences in program setting or region. There were significant differences in program size (*p* = .012) with under-representation of small programs and over-representation of mid-size programs (**Table 1**).

**Table 1:**
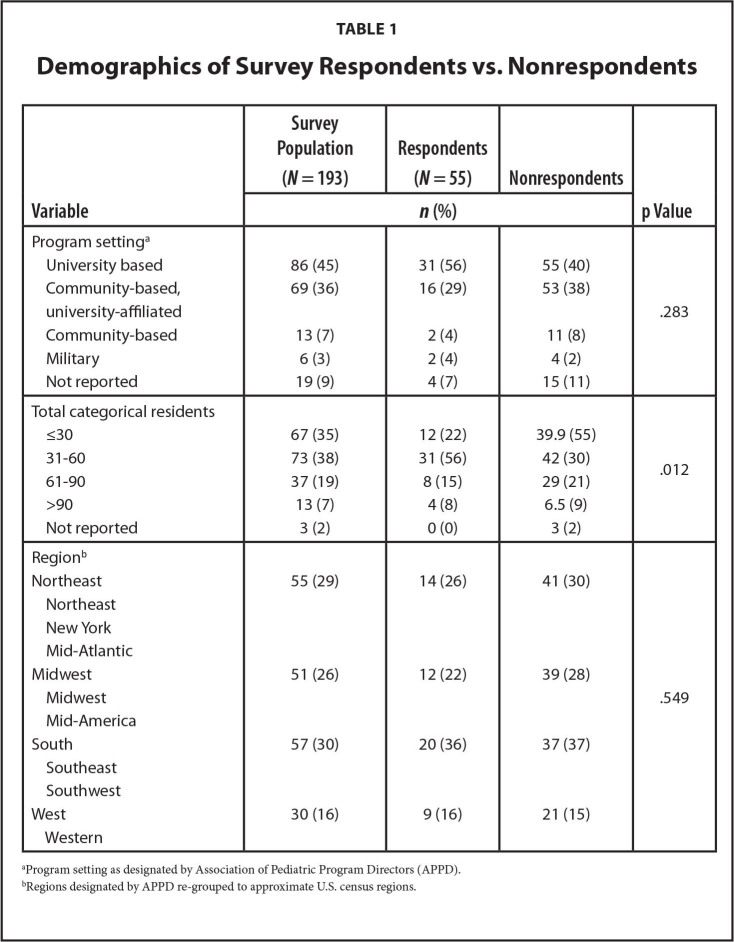
Demographics of Survey Respondents vs. Nonrespondents

**Variable**	**Survey Population (*N* = 193)**	**Respondents (*N* = 55)**	**Nonrespondents**	**p Value**

	***n* (%)**			

Program setting^a^				
University based	86 (45)	31 (56)	55 (40)	.283
Community-based, university-affiliated	69 (36)	16 (29)	53 (38)	
Community-based	13 (7)	2 (4)	11 (8)	
Military	6 (3)	2 (4)	4 (2)	
Not reported	19 (9)	4 (7)	15 (11)	

Total categorical residents				
≤30	67 (35)	12 (22)	39.9 (55)	.012
31-60	73 (38)	31 (56)	42 (30)	
61-90	37 (19)	8 (15)	29 (21)	
>90	13 (7)	4 (8)	6.5 (9)	
Not reported	3 (2)	0 (0)	3 (2)	

Region^b^				
Northeast	55 (29)	14 (26)	41 (30)	
Northeast				
New York				
Mid-Atlantic				
Midwest	51 (26)	12 (22)	39 (28)	
Midwest				.549
Mid-America				
South	57 (30)	20 (36)	37 (37)	
Southeast				
Southwest				
West	30 (16)	9 (16)	21 (15)	
Western				

a Program setting as designated by Association of Pediatric Program Directors (APPD).

b Regions designated by APPD re-grouped to approximate U.S. census regions.

Of responding programs, 51 (93%) strongly agreed or agreed that health literacy concepts are important to include in residency education, and 46 (84%) reported residents would benefit from more training about health literacy. Despite this, only 24 (44%) reported the presence of health literacy education. The majority (58%) of those without known health literacy education were uncertain about future plans to integrate it.

Of programs that reported health literacy education, there was great variability in content, teaching modalities, and evaluation methods. The most commonly taught concepts were the prevalence of (87%) and risk factors for (91%) limited health literacy. Only 30% taught the universal precautions approach, and 48% taught chunking information. The majority (79%) of programs with health literacy education only assess skills implicitly as part of evaluation of resident communication (**Table 2**).

**Table 2:**
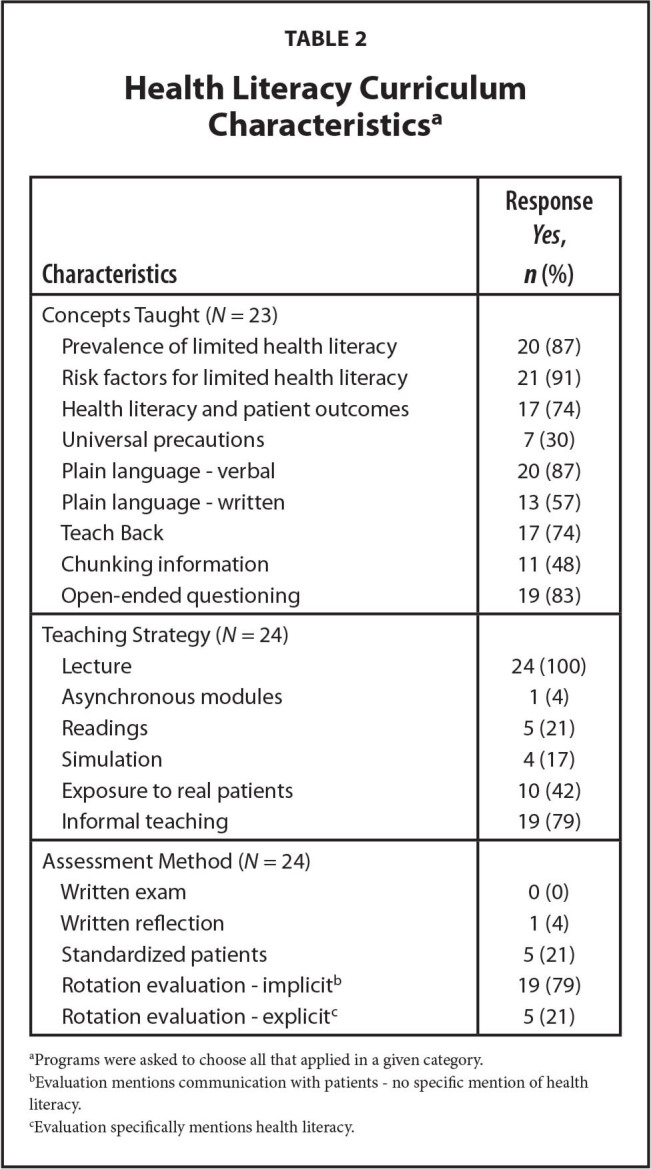
Health Literacy Curriculum Characteristics^a^

**Characteristics**	**Response *Yes*, *n* (%)**

Concepts Taught (*N* = 23)	
Prevalence of limited health literacy	20 (87)
Risk factors for limited health literacy	21 (91)
Health literacy and patient outcomes	17 (74)
Universal precautions	7 (30)
Plain language - verbal	20 (87)
Plain language - written	13 (57)
Teach Back	17 (74)
Chunking information	11 (48)
Open-ended questioning	19 (83)

Teaching Strategy (*N* = 24)	
Lecture	24 (100)
Asynchronous modules	1 (4)
Readings	5 (21)
Simulation	4 (17)
Exposure to real patients	10 (42)
Informal teaching	19 (79)

Assessment Method (*N* = 24)	
Written exam	0 (0)
Written reflection	1 (4)
Standardized patients	5 (21)
Rotation evaluation - implicit^b^	19 (79)
Rotation evaluation - explicit^c^	5 (21)

a Programs were asked to choose all that applied in a given category.

b Evaluation mentions communication with patients - no specific mention of health literacy.

c Evaluation specifically mentions health literacy.

Based on thematic analysis, major barriers to initiating and maintaining health literacy education included time, competing priorities, lack of local expertise, and lack of resources (**Table A**). One APD shared: “The biggest barrier is having formal time and energy dedicated to this topic. This is often mixed in and a part of many things that we do but is not highlighted in and of itself.” Another APD explained: “Probably the biggest barrier is not really understanding the scope of health literacy challenges in various populations or previous successes/failures with physicians attempting to address those impacts.”

**Table A:**
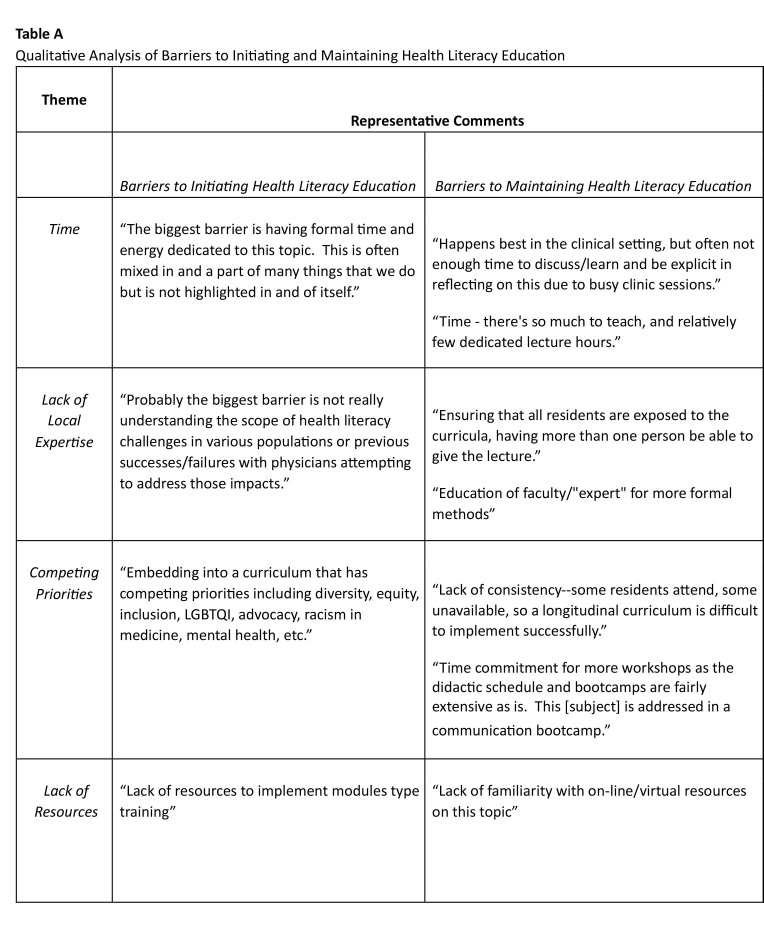
Qualitative Analysis of Barriers to Initiating and Maintaining Health Literacy Education

**Theme**	**Representative Comments**	

	*Barriers to Initiating Health Literacy Education*	*Barriers to Maintaining Health Literacy Education*

*Time*	“The biggest barrier is having formal time and energy dedicated to this topic. This is often mixed in and a part of many things that we do but is not highlighted in and of itself.”	“Happens best in the clinical setting, but often not enough time to discuss/learn and be explicit in reflecting on this due to busy clinic sessions.”
		“Time - there's so much to teach, and relatively few dedicated lecture hours.”

*Lack of Local Expertise*	“Probably the biggest barrier is not really understanding the scope of health literacy challenges in various populations or previous successes/failures with physicians attempting to address those impacts.”	“Ensuring that all residents are exposed to the curricula, having more than one person be able to give the lecture.”
		“Education of faculty/“expert” for more formal methods”

*Competing Priorities*	“Embedding into a curriculum that has competing priorities including diversity, equity, inclusion, LGBTQI, advocacy, racism in medicine, mental health, etc.”	“Lack of consistency--some residents attend, some unavailable, so a longitudinal curriculum is difficult to implement successfully.”
		“Time commitment for more workshops as the didactic schedule and bootcamps are fairly extensive as is. This [subject] is addressed in a communication bootcamp.”

*Lack of Resources*	“Lack of resources to implement modules type training”	“Lack of familiarity with on-line/virtual resources on this topic”

## Discussion

Our study is the first to describe the current state of pediatric resident health literacy education and provides insight into opportunities to introduce health literacy curricula as a vital component of promoting pediatric health equity, safety, quality, and patient/family centered care. While the majority of APDs responding to our survey believed including health literacy concepts in pediatric residency education was important, less than half reported health literacy education in their programs. Multiple barriers to incorporating health literacy education, including lack of time and expertise, were noted, underscoring the need for health literacy champions and readily available curricular resources. Most programs that do teach health literacy are doing so as a one-time didactic or workshop, as opposed to a longitudinal curriculum; this may not be sufficient to ensure optimal skill development and retention (Coleman et al., 2022). Moreover, the majority are not teaching evidence-based health literacy strategies, such as the universal precautions approach to clear communication (AHRQ, 2024), or explicitly assessing outcomes to ensure acquisition and retention of knowledge and skills.

Our findings align with those of previous nonpediatric studies investigating the presence of health literacy education within undergraduate and graduate medical education programs. Coleman et al. (2016) reported a similar large percentage of family medicine residency program leaders who agreed health literacy education is important, but less than half (42%) reported teaching it. Among community-based internal medicine residency programs, only 43% of programs reported any formal health literacy education (Ali, 2013). Interestingly, Coleman and Appy (2012) reported 72% of U.S. allopathic medical schools teach health literacy within their required curricula, but it appears this education is not occurring as frequently in residency, resulting in a persistent skills gap in use of health literacy-informed communication techniques (Glick et al., 2023). All these studies report similar variability in health literacy content, teaching modalities, and evaluation methods (Ali, 2013; Coleman & Appy, 2012; Coleman et al., 2017). Together with our pediatrics-focused study, they highlight the critical gap in health literacy education across the physician education continuum and among various residency programs. This gap has persisted despite evidence as early as 2003 and 2009 that low health literacy is highly prevalent among U.S. adults (Institute of Education Sciences, 2003) and the pediatric parent population (Yin et al., 2009), respectively.

Our study is limited by a low response rate which may make our results difficult to generalize. However, our results likely overestimate the prevalence of health literacy education within pediatric residencies given possible social desirability bias and the demographic breakdown of responding programs. Demographic differences between responding and non-responding programs were significant based on size, with over-representation of mid-size programs. Even among larger programs with potentially more resources, health literacy education prevalence was low. It is possible that APDs may not be aware of health literacy education at their programs, especially if it is embedded within general communication or family centered rounds education, but not specifically identified as health literacy.

Initial steps to address the identified gaps in health literacy education include increasing awareness and dissemination of existing resources, such as those through AHRQ (2024) and MedEdPORTAL (Spengler et al., 2021). Given limited time and competing priorities noted by participants, it may be helpful to more explicitly integrate health literacy practices into already existing residency curricula. Faculty development in didactic and bedside teaching of health literacy informed practices is needed, as many APDs noted lack of faculty expertise as a barrier.

This study is part of a larger initiative on behalf of the APA Health Literacy SIG to develop a health literacy curriculum specific to pediatric residency training. Combined with increased focus on system changes to support application of health literacy principles, and development of standardized health literacy specific assessment tools, this multifaceted approach aligns with Healthy People 2030's focus on organizational health literacy and paves the way for a more equitable health care system (Healthy People 2030, n.d.; Healthy People 2020, n.d.).
